# *catena*-Poly[[[diaqua­copper(II)]-μ-2,2′-{[*p*-phenyl­enebis(oxymethyl­ene)]bis­(pyridinium-3,1-di­yl)}diacetate] dibromide]

**DOI:** 10.1107/S1600536810014510

**Published:** 2010-05-08

**Authors:** Wei-Cheng Pan, Hong-Lei Lian

**Affiliations:** a College of Chemical Engineering and Foods, Zhongzhou University, Zhengzhou, Henan 450044, People’s Republic of China; b College of Chemical Engineering, Zhengzhou University, Zhengzhou, Henan 450001, People’s Republic of China

## Abstract

The title centrosymmetric coordination polymer, {[Cu(C_22_H_20_N_2_O_6_)(H_2_O)_2_]Br_2_}_*n*_, formed by the reaction of the flexible double betaine ligand 2,2′-{[*p*-phenyl­enebis(oxymethyl­ene)]bis­(pyridine-3,1-di­yl)}diacetic acid with CuBr_2_, contains a Cu(II) atom (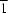 symmetry) which is surrounded by two water molecules and bridged by two anions in a square-planar coordination. In the crystal, polymeric zigzag chains are linked *via* O—H⋯Br inter­actions, forming a two-dimensional network extending parallel to (011).

## Related literature

For double betaine coordination polymers, see: Zhang *et al.* (2004 ▶).
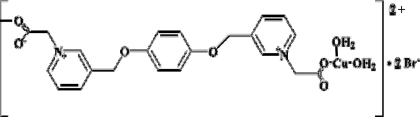


## Experimental

### 

#### Crystal data

[Cu(C_22_H_20_N_2_O_6_)(H_2_O)_2_]Br_2_
*M* *_r_* = 667.78
Triclinic, 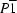

*a* = 7.5422 (7) Å
*b* = 9.3001 (8) Å
*c* = 9.9890 (9) Å
α = 64.194 (2)°
β = 79.405 (2)°
γ = 77.000 (2)°
*V* = 611.66 (10) Å^3^
*Z* = 1
Mo *K*α radiation
μ = 4.21 mm^−1^
*T* = 293 K
0.52 × 0.30 × 0.30 mm

#### Data collection

Bruker SMART CCD area-detector diffractometer
Absorption correction: multi-scan (*SADABS*; Sheldrick, 1996 ▶) *T*_min_ = 0.218, *T*_max_ = 0.365
3313 measured reflections
2129 independent reflections
1746 reflections with *I* > 2σ(*I*)
*R*_int_ = 0.087

#### Refinement

*R*[*F* ^2^ > 2σ(*F* ^2^)] = 0.061
*wR*(*F* ^2^) = 0.163
*S* = 1.03
2129 reflections
160 parameters
3 restraints
H-atom parameters constrained
Δρ_max_ = 0.82 e Å^−3^
Δρ_min_ = −0.95 e Å^−3^

### 

Data collection: *SMART* (Bruker, 2007 ▶); cell refinement: *SAINT* (Bruker, 2007 ▶); data reduction: *SAINT*; program(s) used to solve structure: *SHELXS97* (Sheldrick, 2008 ▶); program(s) used to refine structure: *SHELXL97* (Sheldrick, 2008 ▶); molecular graphics: *SHELXTL* (Sheldrick, 2008 ▶); software used to prepare material for publication: *SHELXTL*.

## Supplementary Material

Crystal structure: contains datablocks I, global. DOI: 10.1107/S1600536810014510/su2168sup1.cif

Structure factors: contains datablocks I. DOI: 10.1107/S1600536810014510/su2168Isup2.hkl

Additional supplementary materials:  crystallographic information; 3D view; checkCIF report

## Figures and Tables

**Table 1:** Hydrogen-bond geometry (Å, °)

*D*—H⋯*A*	*D*—H	H⋯*A*	*D*⋯*A*	*D*—H⋯*A*
O1*W*—H1*WB*⋯Br1	0.52	2.74	3.220 (9)	155
O1*W*—H1*WA*⋯Br1^i^	0.78	2.38	3.139 (9)	167

Symmetry code: (i) 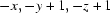.

**Fig. 1.**
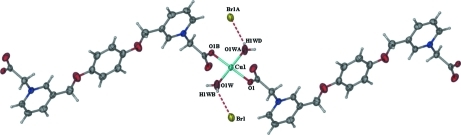
A portion of the infinite chain of the title compound, with atom labels and 50% probability displacement ellipsoids (Symmetry codes: A: -x+2, -y+2, -z+2; B: -x+1, -y+1, -z+1).

**Fig. 2.**
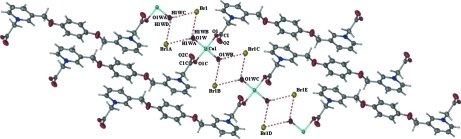
A view of the two-dimensional network formed as a result of the O—H···Br hydrogen bonds (dashed lines); Symmetry codes: A: -x, -y + 1, -z + 1; B: -x + 1, -y + 1, -z + 1; C: x + 1, y, z; D: -x + 2, -y + 1, -z + 1; D: x + 2, y, z.

## supplementary crystallographic information

### Comment

Supramolecular assembly is a powerful tool in the design of metallo-organic based complex molecular architectures. Ligands of the double betaine type are known to be generators of variable coordination frameworks in organic-inorganic hybrid materials (Zhang *et al.*, 2004).

The title complex is centrosymmetric and exists as infinite zigzag chains (Fig. 1). In the crystal the bromide serves as a hydrogen-bond acceptor and the coordinated water molecules as donors leading to the formation of a two-dimensional network (Fig. 2 and Table 1).

### Experimental

An aqueous solution (5 ml of H_2_O) of 1,4-bis(3-picolyloxyl)benzene- *N*,*N*^'^-diacetate) [0.08 g, 0.2 mmol] and CuBr_2_ (0.067 g, 0.3 mmol) were mixed together and heated at 340 K for 10 min with continuous stirring. The mixture was then filtered and upon slow evaporation of the filtrate, at RT for several weeks, blue block-shaped crystals were obtained (Yield ca. 58% based on L).

### Refinement

The water H-atoms were located in a difference electron-density map and were held fixed with *U*_iso_(H) = 1.5*U*_eq_(O). The C-bound H-atoms were positioned geometrically and refined using a riding model: C—H = 0.93–0.97 Å and with *U*_iso_(H) = 1.2*U*_eq_(C).

### Crystal data

**Table:**

[Cu(C_22_H_20_N_2_O_6_)(H_2_O)_2_]Br_2_	*Z* = 1
*M**_r_* = 667.78	*F*(000) = 333
Triclinic, *P*1	*D*_x_ = 1.813 Mg m^−^^3^
Hall symbol: -P 1	Mo *K*α radiation, λ = 0.71073 Å
*a* = 7.5422 (7) Å	Cell parameters from 126 reflections
*b* = 9.3001 (8) Å	θ = 2.0–27.5°
*c* = 9.9890 (9) Å	µ = 4.21 mm^−^^1^
α = 64.194 (2)°	*T* = 293 K
β = 79.405 (2)°	Block, blue
γ = 77.000 (2)°	0.52 × 0.30 × 0.30 mm
*V* = 611.66 (10) Å^3^	

### Data collection

**Table:**

Bruker SMART CCD area-detector diffractometer	2129 independent reflections
Radiation source: fine-focus sealed tube	1746 reflections with *I* > 2σ(*I*)
graphite	*R*_int_ = 0.087
phi and ω scans	θ_max_ = 25.0°, θ_min_ = 2.3°
Absorption correction: multi-scan (*SADABS*; Sheldrick, 1996)	*h* = −8→8
*T*_min_ = 0.218, *T*_max_ = 0.365	*k* = −10→11
3313 measured reflections	*l* = −10→11

### Refinement

**Table:**

Refinement on *F*^2^	Primary atom site location: structure-invariant direct methods
Least-squares matrix: full	Secondary atom site location: difference Fourier map
*R*[*F*^2^ > 2σ(*F*^2^)] = 0.061	Hydrogen site location: inferred from neighbouring sites
*wR*(*F*^2^) = 0.163	H-atom parameters constrained
*S* = 1.03	*w* = 1/[σ^2^(*F*_o_^2^) + (0.1116*P*)^2^] where *P* = (*F*_o_^2^ + 2*F*_c_^2^)/3
2129 reflections	(Δ/σ)_max_ < 0.001
160 parameters	Δρ_max_ = 0.82 e Å^−^^3^
3 restraints	Δρ_min_ = −0.95 e Å^−^^3^

### Special details

**Table:**

Geometry. All esds (except the esd in the dihedral angle between two l.s. planes) are estimated using the full covariance matrix. The cell esds are taken into account individually in the estimation of esds in distances, angles and torsion angles; correlations between esds in cell parameters are only used when they are defined by crystal symmetry. An approximate (isotropic) treatment of cell esds is used for estimating esds involving l.s. planes.
Refinement. Refinement of *F*^2^ against ALL reflections. The weighted *R*-factor wR and goodness of fit *S* are based on *F*^2^, conventional *R*-factors *R* are based on *F*, with *F* set to zero for negative *F*^2^. The threshold expression of *F*^2^ > σ(*F*^2^) is used only for calculating *R*-factors(gt) etc. and is not relevant to the choice of reflections for refinement. *R*-factors based on *F*^2^ are statistically about twice as large as those based on *F*, and *R*- factors based on ALL data will be even larger.

### Fractional atomic coordinates and isotropic or equivalent isotropic displacement parameters (Å^2^)

**Table:**

	*x*	*y*	*z*	*U*_iso_*/*U*_eq_	
Cu1	0.5000	0.5000	0.5000	0.0266 (4)	
Br1	−0.09111 (11)	0.71414 (10)	0.61132 (10)	0.0434 (4)	
O1	0.4292 (7)	0.6175 (6)	0.6239 (5)	0.0288 (12)	
O1W	0.2454 (8)	0.4936 (11)	0.5040 (10)	0.089 (3)	
H1WA	0.1963	0.4563	0.4678	0.133*	
H1WB	0.1830	0.5087	0.5306	0.133*	
O2	0.4295 (9)	0.8338 (7)	0.4071 (6)	0.0462 (16)	
O3	0.7158 (10)	0.8939 (9)	0.9512 (7)	0.0570 (19)	
N1	0.3377 (9)	0.7917 (7)	0.7897 (7)	0.0290 (14)	
C1	0.4042 (10)	0.7683 (9)	0.5435 (8)	0.0293 (16)	
C2	0.3214 (11)	0.8765 (9)	0.6271 (8)	0.0315 (17)	
H2A	0.3826	0.9702	0.5861	0.038*	
H2B	0.1932	0.9148	0.6107	0.038*	
C3	0.2186 (11)	0.6898 (10)	0.8751 (9)	0.0347 (18)	
H3A	0.1288	0.6746	0.8321	0.042*	
C4	0.2320 (11)	0.6095 (10)	1.0252 (9)	0.0375 (19)	
H4A	0.1528	0.5373	1.0848	0.045*	
C5	0.3618 (12)	0.6351 (10)	1.0880 (9)	0.0381 (19)	
H5A	0.3681	0.5819	1.1907	0.046*	
C6	0.4849 (11)	0.7399 (9)	1.0003 (8)	0.0313 (17)	
C7	0.4693 (11)	0.8171 (9)	0.8479 (8)	0.0292 (16)	
H7A	0.5501	0.8867	0.7856	0.035*	
C8	0.6243 (13)	0.7737 (11)	1.0664 (10)	0.043 (2)	
H8A	0.5654	0.8110	1.1431	0.051*	
H8B	0.7109	0.6759	1.1117	0.051*	
C9	0.8556 (11)	0.9412 (11)	0.9825 (10)	0.0377 (19)	
C10	0.9060 (12)	0.8939 (11)	1.1252 (9)	0.040 (2)	
H10A	0.8431	0.8239	1.2087	0.047*	
C11	0.9504 (12)	1.0480 (11)	0.8589 (9)	0.039 (2)	
H11A	0.9157	1.0805	0.7640	0.047*	

### Atomic displacement parameters (Å^2^)

**Table:**

	*U*^11^	*U*^22^	*U*^33^	*U*^12^	*U*^13^	*U*^23^
Cu1	0.0268 (7)	0.0331 (8)	0.0283 (7)	−0.0080 (5)	−0.0023 (5)	−0.0189 (6)
Br1	0.0384 (6)	0.0471 (6)	0.0532 (7)	−0.0103 (4)	−0.0074 (4)	−0.0258 (5)
O1	0.032 (3)	0.034 (3)	0.026 (3)	−0.006 (2)	−0.002 (2)	−0.018 (2)
O1W	0.028 (3)	0.140 (8)	0.172 (9)	−0.013 (4)	0.001 (4)	−0.136 (8)
O2	0.077 (5)	0.041 (3)	0.021 (3)	−0.016 (3)	0.001 (3)	−0.012 (2)
O3	0.063 (4)	0.075 (5)	0.038 (4)	−0.035 (4)	−0.015 (3)	−0.013 (3)
N1	0.036 (3)	0.028 (3)	0.027 (3)	−0.006 (3)	−0.001 (3)	−0.014 (3)
C1	0.031 (4)	0.032 (4)	0.032 (4)	−0.010 (3)	−0.005 (3)	−0.017 (3)
C2	0.042 (4)	0.025 (4)	0.028 (4)	−0.008 (3)	−0.005 (3)	−0.010 (3)
C3	0.035 (4)	0.037 (4)	0.037 (4)	−0.008 (3)	−0.002 (3)	−0.019 (4)
C4	0.038 (5)	0.041 (5)	0.031 (4)	−0.015 (4)	0.005 (3)	−0.012 (4)
C5	0.045 (5)	0.039 (4)	0.022 (4)	−0.002 (4)	−0.002 (3)	−0.007 (3)
C6	0.036 (4)	0.033 (4)	0.028 (4)	0.001 (3)	−0.007 (3)	−0.017 (3)
C7	0.035 (4)	0.026 (4)	0.029 (4)	−0.007 (3)	−0.003 (3)	−0.013 (3)
C8	0.050 (5)	0.047 (5)	0.034 (4)	−0.006 (4)	−0.011 (4)	−0.018 (4)
C9	0.036 (4)	0.046 (5)	0.042 (5)	−0.001 (4)	−0.014 (4)	−0.026 (4)
C10	0.042 (5)	0.042 (5)	0.035 (4)	−0.003 (4)	−0.004 (4)	−0.018 (4)
C11	0.041 (5)	0.053 (5)	0.032 (4)	−0.004 (4)	−0.012 (4)	−0.023 (4)

### Geometric parameters (Å, °)

**Table:**

Cu1—O1^i^	1.919 (5)	C3—H3A	0.9300
Cu1—O1	1.919 (5)	C4—C5	1.368 (12)
Cu1—O1W	1.927 (6)	C4—H4A	0.9300
Cu1—O1W^i^	1.927 (6)	C5—C6	1.394 (12)
O1—C1	1.266 (9)	C5—H5A	0.9300
O1W—H1WA	0.7774	C6—C7	1.387 (11)
O1W—H1WB	0.5183	C6—C8	1.492 (12)
O2—C1	1.223 (9)	C7—H7A	0.9300
O3—C9	1.361 (11)	C8—H8A	0.9700
O3—C8	1.409 (11)	C8—H8B	0.9700
N1—C7	1.348 (10)	C9—C11	1.396 (12)
N1—C3	1.353 (10)	C9—C10	1.396 (12)
N1—C2	1.480 (9)	C10—C11^ii^	1.376 (13)
C1—C2	1.535 (10)	C10—H10A	0.9300
C2—H2A	0.9700	C11—C10^ii^	1.376 (13)
C2—H2B	0.9700	C11—H11A	0.9300
C3—C4	1.365 (11)		
			
O1^i^—Cu1—O1	180	C3—C4—H4A	119.9
O1^i^—Cu1—O1W	91.4 (2)	C5—C4—H4A	119.9
O1—Cu1—O1W	88.6 (2)	C4—C5—C6	120.9 (7)
O1^i^—Cu1—O1W^i^	88.6 (2)	C4—C5—H5A	119.5
O1—Cu1—O1W^i^	91.4 (2)	C6—C5—H5A	119.5
O1W—Cu1—O1W^i^	180	C7—C6—C5	117.4 (7)
C1—O1—Cu1	110.1 (5)	C7—C6—C8	120.6 (7)
Cu1—O1W—H1WA	131.9	C5—C6—C8	122.0 (7)
Cu1—O1W—H1WB	138.6	N1—C7—C6	120.3 (7)
H1WA—O1W—H1WB	89.3	N1—C7—H7A	119.8
C9—O3—C8	119.4 (7)	C6—C7—H7A	119.8
C7—N1—C3	122.2 (7)	O3—C8—C6	108.1 (7)
C7—N1—C2	119.7 (6)	O3—C8—H8A	110.1
C3—N1—C2	118.1 (7)	C6—C8—H8A	110.1
O2—C1—O1	126.5 (7)	O3—C8—H8B	110.1
O2—C1—C2	117.8 (7)	C6—C8—H8B	110.1
O1—C1—C2	115.6 (6)	H8A—C8—H8B	108.4
N1—C2—C1	112.9 (6)	O3—C9—C11	115.3 (7)
N1—C2—H2A	109.0	O3—C9—C10	125.3 (8)
C1—C2—H2A	109.0	C11—C9—C10	119.4 (8)
N1—C2—H2B	109.0	C11^ii^—C10—C9	119.3 (8)
C1—C2—H2B	109.0	C11^ii^—C10—H10A	120.4
H2A—C2—H2B	107.8	C9—C10—H10A	120.4
N1—C3—C4	119.1 (8)	C10^ii^—C11—C9	121.3 (8)
N1—C3—H3A	120.5	C10^ii^—C11—H11A	119.3
C4—C3—H3A	120.5	C9—C11—H11A	119.3
C3—C4—C5	120.1 (8)		
			
O1^i^—Cu1—O1—C1	112 (100)	C4—C5—C6—C8	178.2 (8)
O1W—Cu1—O1—C1	−91.2 (6)	C3—N1—C7—C6	−1.1 (11)
O1W^i^—Cu1—O1—C1	88.8 (6)	C2—N1—C7—C6	179.3 (7)
Cu1—O1—C1—O2	−3.7 (10)	C5—C6—C7—N1	0.9 (11)
Cu1—O1—C1—C2	171.9 (5)	C8—C6—C7—N1	−176.9 (7)
C7—N1—C2—C1	101.3 (8)	C9—O3—C8—C6	−177.3 (7)
C3—N1—C2—C1	−78.4 (9)	C7—C6—C8—O3	2.8 (11)
O2—C1—C2—N1	−166.3 (7)	C5—C6—C8—O3	−175.0 (7)
O1—C1—C2—N1	17.8 (9)	C8—O3—C9—C11	173.5 (8)
C7—N1—C3—C4	−0.1 (12)	C8—O3—C9—C10	−7.8 (13)
C2—N1—C3—C4	179.5 (7)	O3—C9—C10—C11^ii^	−179.5 (9)
N1—C3—C4—C5	1.4 (12)	C11—C9—C10—C11^ii^	−0.8 (14)
C3—C4—C5—C6	−1.5 (13)	O3—C9—C11—C10^ii^	179.6 (8)
C4—C5—C6—C7	0.4 (12)	C10—C9—C11—C10^ii^	0.8 (14)

Symmetry codes: (i) −*x*+1, −*y*+1, −*z*+1; (ii) −*x*+2, −*y*+2, −*z*+2.

### Hydrogen-bond geometry (Å, °)

**Table:**

*D*—H···*A*	*D*—H	H···*A*	*D*···*A*	*D*—H···*A*
O1W—H1WB···Br1	0.52	2.74	3.220 (9)	155
O1W—H1WA···Br1^iii^	0.78	2.38	3.139 (9)	167

Symmetry codes:  (iii) −*x*, −*y*+1, −*z*+1.
